# Acceptance of Virtual Reality in Trainees Using a Technology Acceptance Model: Survey Study

**DOI:** 10.2196/60767

**Published:** 2024-12-23

**Authors:** Ellen Y Wang, Daniel Qian, Lijin Zhang, Brian S-K Li, Brian Ko, Michael Khoury, Meghana Renavikar, Avani Ganesan, Thomas J Caruso

**Affiliations:** 1 Department of Anesthesiology, Perioperative and Pain Medicine, Stanford University School of Medicine, Palo Alto, CA, United States; 2 Department of Medical Education, Icahn School of Medicine at Mount Sinai, New York, NY, United States; 3 Department of Developmental and Psychological Science, Stanford University Graduate School of Education, Palo Alto, CA, United States; 4 Princeton University, Princeton, NJ, United States; 5 University of California, Berkeley, Berkeley, CA, United States; 6 Stanford Chariot Program, Lucile Packard Children’s Hospital Stanford, Stanford, CA, United States; 7 California Northstate University College of Medicine, Elk Grove, CA, United States

**Keywords:** virtual reality, technology assessment, graduate medical education trainees, medical education, technology adoption, Technology Acceptance Model, factor analysis, VR, TAM, United Theory of Acceptance and Use of Technology, UTAUT

## Abstract

**Background:**

Virtual reality (VR) technologies have demonstrated therapeutic usefulness across a variety of health care settings. However, graduate medical education (GME) trainee perspectives on VR acceptability and usability are limited. The behavioral intentions of GME trainees with regard to VR as an anxiolytic tool have not been characterized through a theoretical framework of technology adoption.

**Objective:**

The primary aim of this study was to apply a hybrid Technology Acceptance Model (TAM) and a United Theory of Acceptance and Use of Technology (UTAUT) model to evaluate factors that predict the behavioral intentions of GME trainees to use VR for patient anxiolysis. The secondary aim was to assess the reliability of the TAM-UTAUT.

**Methods:**

Participants were surveyed in June 2023. GME trainees participated in a VR experience used to reduce perioperative anxiety. Participants then completed a survey evaluating demographics, perceptions, attitudes, environmental factors, and behavioral intentions that influence the adoption of new technologies.

**Results:**

In total, 202 of 1540 GME trainees participated. Only 198 participants were included in the final analysis (12.9% participation rate). Perceptions of usefulness, ease of use, and enjoyment; social influence; and facilitating conditions predicted intention to use VR. Age, past use, price willing to pay, and curiosity were less strong predictors of intention to use. All confirmatory factor analysis models demonstrated a good fit. All domain measurements demonstrated acceptable reliability.

**Conclusions:**

This TAM-UTAUT demonstrated validity and reliability for predicting the behavioral intentions of GME trainees to use VR as a therapeutic anxiolytic in clinical practice. Social influence and facilitating conditions are modifiable factors that present opportunities to advance VR adoption, such as fostering exposure to new technologies and offering relevant training and social encouragement. Future investigations should study the model’s reliability within specialties in different geographic locations.

## Introduction

The Technology Acceptance Model (TAM) is the leading theoretical framework for evaluating consumer adoption of new technologies [1-3]. The model assesses variables such as perceptions of usefulness, ease of use, and attitudes toward technologies as indicators for intention to use [1,4]. Since the original 1989 model, TAMs have been applied across a wide range of technologies and with high predictive reliability. In 2010, a TAM successfully predicted factors indicative of health informatics acceptance [2]. In the last several years, the TAM framework has characterized behaviors and attitudes toward several health care innovations, including digital health services, contact tracing during the COVID-19 pandemic, and adverse event reporting systems [5-8].

Virtual reality (VR) is a new class of technologies involving head-mounted devices that have a variety of health care applications, including as a therapeutic adjunct for anxiolysis and as an educational tool for medical training [9-20]. A modified TAM assessed consumers’ perceived enjoyment, age, curiosity, past use, and willingness to pay for VR as an anxiolytic adjunct [21]. In 2023, this VR TAM was applied to pediatric health care clinicians with strong validity and high reliability [22]. Beyond the conventional variables of the TAM, the United Theory of Acceptance and Use of Technology (UTAUT) model adds socioenvironmental variables such as social influence and facilitating conditions as predictive factors [23]. UTAUT proponents believe that the lack of these factors in conventional TAMs limits their generalizability and instead opt for models that also include UTAUT social variables [23,24].

Despite VR’s therapeutic usefulness, widespread clinical adoption is lacking. Barriers include lack of technical skills, organizational cultures that are slow to adopt new technologies, and perceived usefulness to care [25,26]. Because residency and fellowship experiences influence future patient care delivery, understanding perceptions of VR as adjunct therapy is important in early career professionals, such as graduate medical education (GME) trainees [27]. While GME trainees lack the institutional influence or purchasing power to act upon the intention to use and intention to purchase variables assessed by the TAM framework, it is important to understand which factors affect these intentions because members of this generation of physicians may provide practice-level decision-making input in the future. Additionally, identifying factors associated with technology adoption creates opportunity for sustainable and effective implementation [28]. However, GME trainee perspectives on VR acceptability and usability are limited. The behavioral intentions of GME trainees with regard to VR as an anxiolytic tool have not been characterized through a theoretical framework of technology adoption.

Given the importance VR will play in future health care delivery, a hybrid TAM-UTAUT that predicts use among GME trainees was developed. The primary aim modeled factors that predict the behavioral intentions of GME trainees’ use of VR as an anxiolytic adjunct. The secondary aim assessed the reliability of the TAM-UTAUT measurements for modeling use in the health care context.

## Methods

### Context and Setting

This study was conducted in 2023 at Stanford Hospital and Lucile Packard Children’s Hospital Stanford (Stanford University). Oculus Go (Meta, Inc) VR headsets displayed the application Pebbles the Penguin (Stanford Chariot Program). This third-person perspective application displays a head-controlled cartoon penguin sliding down a mountain collecting colorful objects for points. The application is intuitive, continues in perpetuity, and is successfully used to reduce perioperative and procedural anxiety [10,29,30].

The Stanford GME department includes over 1000 residents and fellows and over 100 training programs. The inclusion criteria were postgraduate medical trainees in any year of a Stanford residency or fellowship program. Exclusion criteria were individuals with nausea, motion sickness, seizure disorders, and active illness. With a significance level of α=.05, it was determined that a minimum sample size of 174 participants would be needed to test the study hypotheses.

### Ethical Considerations

The Stanford University Internal Review Board approved a waiver of consent. Trainees provided their informed consent prior to participation. All participants were informed of their ability to opt out of the study without consequence. No financial payments were provided for participation. No personal identifying information was collected as part of this study.

### Intervention

Trained research assistants (RAs), including coauthors BSKL, BK, MK, MR, and AG, conducted convenience sampling by recruiting volunteer participants in resident and fellow break rooms, outside cafeterias, and in patient care areas of the hospitals. Upon enrollment, RAs provided participants with descriptions of the clinical use of the VR experience as well as gameplay instructions. Participants completed a demographic survey before playing Pebbles the Penguin for 2 minutes. The application was initiated by the RAs to create a standardized experience for all participants. Following the VR experience, participants completed a survey adapted for health care professionals derived from previous VR TAM or UTAUT models (Multimedia Appendix 1) [21-23].

### Hypotheses

**Figure 1.**
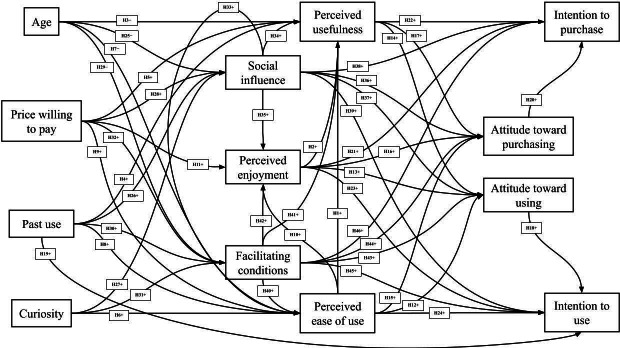
Virtual reality Technology Acceptance Model and United Theory of Acceptance and Use of Technology hypothesis model (adapted from [21-23]). H: hypothesis.

This TAM-UTAUT applied a validated hypothesis model exploring the intention to use VR [21,22]. Perceived ease of use, perceived usefulness, and perceived enjoyment were established in the earliest versions of the TAM and are widely applicable to technology acceptance, as Manis and Choi [21] revalidated in their VR hardware acceptance model in 2019. Perceptions regarding usefulness, ease of use, and enjoyment positively influence attitudes and behaviors toward purchasing and use [21]. These hypotheses stem from the idea that positive perceptions regarding new technologies promote decreased resistance to change [8,21]. Age is hypothesized to negatively influence perceptions surrounding technologies because age is often negatively associated with perceived future opportunities, limiting motivation for adoption [31]. Other demographic variables such as past use and price willing to pay are hypothesized to positively influence perceptions and intent to use. Curiosity, established as an inherent drive to seek novelty, is a positive indicator for perceived ease of use. Individuals with greater innate curiosity may engage with new technologies more out of interest rather than deprivation [21]. From the UTAUT model, social influence and facilitating conditions are both expected to have positive influential effects on perceptions, attitudes, and behavioral intentions (Figure 1) [23]. Social influence can result in a positive impact when adopting popular technologies that favor herd behavior and conformity over limited personal experiences [5,24]. Facilitating conditions are proposed to influence adoption through an organization’s infrastructure support of the proposed technology [5,24].

### Outcomes

The primary outcome was to determine the predictors and validity of the TAM-UTAUT for the adoption of VR as an anxiolytic tool for patient care among a heterogeneous group of GME trainees. The secondary outcome explored the reliability of the model in the health care setting.

### Measures

Demographic data collected prior to the intervention included age, sex, race, ethnicity, specialty, years of trainee experience, and prior VR use. The TAM-UTAUT surveyed aspects of perceived usefulness, perceived ease of use, perceived enjoyment, intention to use, intention to purchase, curiosity, attitude toward using, attitude toward purchasing, and price willing to pay. The TAM-UTAUT also included 2 socioenvironmental variables—social influence and facilitating conditions—to address previous limitations of conventional TAMs (Multimedia Appendix 2).

Price willing to pay was measured on a continuous scale from US $0 to US $1500. Attitudes toward using and purchasing were each measured using 5 questions graded on a 5-point sliding scale. Perceived usefulness and perceived ease of use were each measured using 5 questions graded on a 1‐5 Likert scale, ranging from 1=strongly disagree, 2=mostly disagree, 3=neither agree nor disagree, 4=mostly agree, and 5=strongly agree. The remaining variables were each measured using 4 questions graded on the same Likert scale (Multimedia Appendix 1). Study data were collected, encrypted, and managed securely using REDCap (Research Electronic Data Capture) tools hosted at Stanford University [32,33].

### Analysis

All elements were analyzed as continuous variables. To assess the predictive validity of the TAM-UTAUT, means and SDs were evaluated for each scale. Confirmatory factor analysis (CFA) was conducted using Mplus (version 8.6; Muthén & Muthén, 1998‐2022) to test the construct validity of different scales. Items over 0.7 were considered satisfactory [34,35]. Each item was tested for a normal distribution. For scales that were normally distributed, the maximum likelihood estimation method was used. For nonnormal scales, a robust maximum likelihood method was adopted to estimate the CFA model. The comparative fit index (CFI), root mean square error of approximation (RMSEA), Tucker-Lewis index (TLI), and standardized root mean square residual (SRMR) evaluated model fit. The CFI and TLI indices were accepted above 0.9 [36,37]. The RMSEA and SRMR below 0.08 indicated an acceptable fit [38,39]. The results were interpreted as the difference between each nonreference group.

To assess the secondary outcome of this TAM-UTAUT’s internal consistency and reliability, each scale was evaluated using Cronbach α and composite reliability. Composite reliability is similar to Cronbach α except that it does not assume each item to be equally weighted and accounts for actual factor loadings. Values were considered acceptable if greater than .6 [40].

## Results

### Demographics

In total, 202 of 1540 GME trainees participated (13.1% participation rate). After excluding responses with missing values, 198 participants (12.9% participation rate) were included in the final data analysis (Table 1). The ratio of female to male respondents was 1:1. The average age of all participants was 31.3 (SD 3.5) years, ranging from 23 to 45 years. Among the participants, 72 (36.4%) had no prior exposure to VR, 107 (54%) had 1‐5 experiences with VR, and 19 (9.6%) had 6 or more experiences with VR. The mean number of previous VR exposures was 2.7 (SD 4.5). In total, 44 unique specialties and fellowship subspecialties were represented among the participants. The most frequently represented specialties included pediatrics (n=32, 16.2%), internal medicine (n=27, 13.6%), and pathology (n=20, 10.1%; Multimedia Appendix 3).

**Table 1.** Participant demographics.

Characteristic	Values
Age (years), mean (SD)	31.3 (3.5)
Sex, n (%)	
Male	99 (50)
Female	99 (50)
Race^a^, n (%)	
American Indian or Alaskan Native	0 (0)
Asian	64 (32.3)
Black or African American	11 (5.6)
Native Hawaiian or other Pacific	0 (0)
White	112 (56.6)
More than 1 race	5 (2.5)
Prefer not to answer	6 (3)
Ethnicity, n (%)	
Hispanic or Latino	25 (12.6)
Non-Hispanic or Latino	170 (85.9)
Unknown or chose not to disclose	3 (1.5)
Level of training, n (%)	
PGY1^b^	57 (28.8)
PGY2	34 (17.2)
PGY3	27 (13.6)
PGY4	21 (10.6)
PGY5	27 (13.6)
PGY6 or higher	32 (16.2)
Previous exposure to VR^c^, n (%)	
0 times	72 (36.4)
1‐5 times	107 (54)
6‐9 times	2 (1)
10‐14 times	8 (4)
15‐19 times	1 (0.5)
≥20 times	8 (4)

a Multiple answers allowed.

b PGY: postgraduate year.

c VR: virtual reality.

### Primary Outcome

For each composite domain, mean (SD) of perceived usefulness were 3.498 (0.883), perceived ease of use 3.911 (0.803), perceived enjoyment 4.349 (0.721), intention to use 3.460 (1.064), intention to purchase 3.446 (0.880), curiosity 3.404 (0.908), attitude toward using 3.979 (0.751), attitude toward purchasing 3.810 (0.801), social influence 2.910 (0.729), and facilitating conditions 3.684 (0.707). The mean price willing to pay was US $781.36 (SD US $375.50; Multimedia Appendix 4). All responses demonstrated normality, and the maximum likelihood method estimated the models.

Standardized factor loadings were greater than 0.7 for all items within the perceived usefulness, perceived ease of use, perceived enjoyment, intention to use, attitude toward using, and attitude toward purchasing domains. A total of 2 survey items within intention to purchase, 1 item within curiosity, 2 items within social influence, and 1 item with facilitating conditions were below 0.7 (Multimedia Appendix 5). All CFA models, which take into account all survey items within each scale, demonstrated good fit (CFI 0.984‐1; TLI 0.968‐1; RMSEA 0-0.137; SRMR 0-0.021; Multimedia Appendix 6).

**Figure 2.**
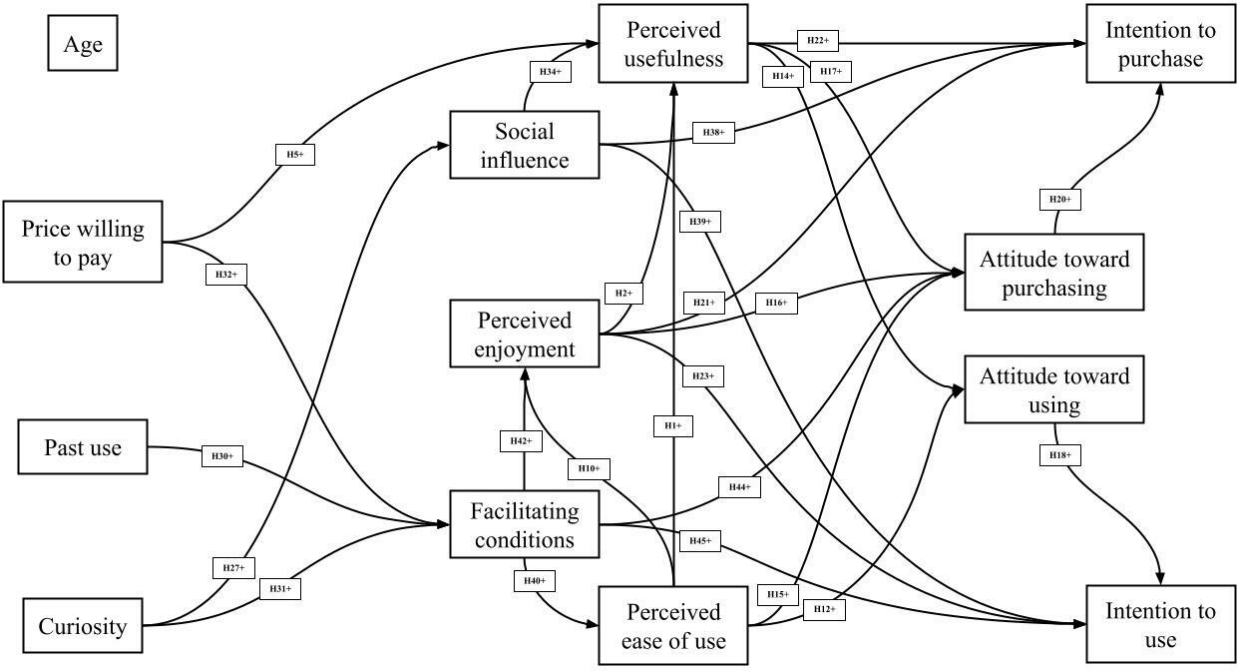
Technology Acceptance Model and United Theory of Acceptance and Use of Technology results—significant estimates. H: hypothesis.

Detailed estimates of the path coefficients were calculated (Figures2  3 and Table 2). Most of the relationships between the perception, attitude, and intention domains were significant, except for the influence of perceived enjoyment on attitude toward using and perceived ease of use on intention to use. Perceived ease of use influenced perceived usefulness (*P*<.001), perceived enjoyment (*P*<.001), attitude toward using (*P*=.04), and attitude toward purchasing (*P*=.03). Perceived enjoyment influenced perceived usefulness (*P*<.001), attitude toward purchasing (*P*=.04), intention to purchase (*P*=.001), and intention to use (*P*<.001). Perceived usefulness influenced attitude toward using (*P*<.001), attitude toward purchasing (*P*<.001), and intention to purchase (*P*<.001; Figure 2 and Table 2).

**Figure 3.**
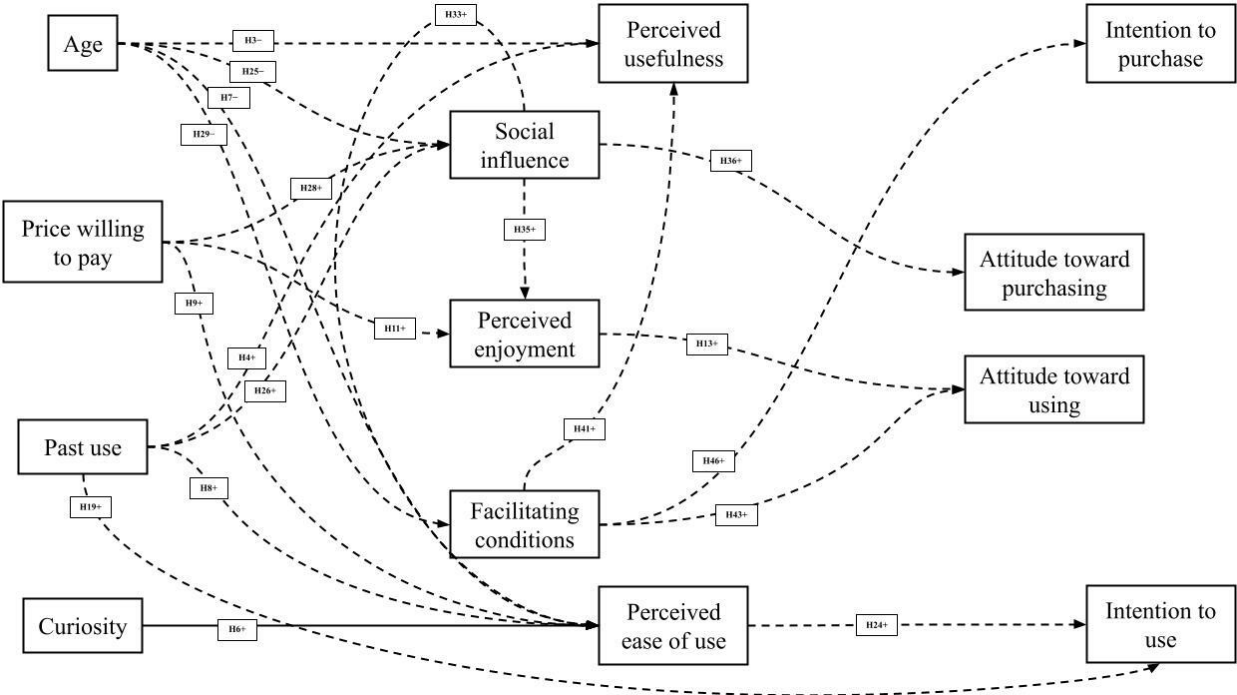
Technology Acceptance Model and United Theory of Acceptance and Use of Technology results—nonsignificant estimates. H: hypothesis.

**Table 2.** Standardized estimates of the Technology Acceptance Model and United Theory of Acceptance and Use of Technology.

	Predictor	Outcome	β (95% CI)	*P* value
H1^a^	Perceived ease of use	Perceived usefulness	.258^b^ (.135 to .381)	<.001
H2	Perceived enjoyment	Perceived usefulness	.299^b^ (.169 to .430)	<.001
H3	Age	Perceived usefulness	−.082 (−.182 to .18)	.11
H4	Past use	Perceived usefulness	.058 (−.042 to .158)	.26
H5	Price willing to pay	Perceived usefulness	.147^b^ (.045 to .250)	.005
H6	Curiosity	Perceived ease of use	−.053 (−.187 to .81)	.44
H7	Age	Perceived ease of use	−.054 (−.179 to .072)	.40
H8	Past use	Perceived ease of use	.067 (−.058 to .193)	.29
H9	Price willing to pay	Perceived ease of use	.034 (−.096 to .164)	.61
H10	Perceived ease of use	Perceived enjoyment	.427^b^ (.318 to .535)	<.001
H11	Price willing to pay	Perceived enjoyment	.035 (−.072 to .142)	.52
H12	Perceived ease of use	Attitude toward using	.146^c^ (.005 to .287)	.04
H13	Perceived enjoyment	Attitude toward using	.104 (−.048 to .256)	.18
H14	Perceived usefulness	Attitude toward using	.276^b^ (.130 to .422)	<.001
H15	Perceived ease of use	Attitude toward purchase	.157^c^ (.012 to .302)	.03
H16	Perceived enjoyment	Attitude toward purchase	−.162^c^ (−.318 to −.006)	.04
H17	Perceived usefulness	Attitude toward purchase	.369^b^ (.221 to .517)	<.001
H18	Attitude toward using	Intention to use	.316^b^ (.205 to .426)	<.001
H19	Past use	Intention to use	−.032 (−.122 to .058)	.49
H20	Attitude toward purchase	Intention to purchase	.309^b^ (.204 to .414)	<.001
H21	Perceived enjoyment	Intention to purchase	.199^b^ (.080 to .318)	.001
H22	Perceived usefulness	Intention to purchase	.243^b^ (.107 to .380)	<.001
H23	Perceived enjoyment	Intention to use	.273^b^ (.149 to .397)	<.001
H24	Perceived ease of use	Intention to use	.089 (−.028 to .207)	.14
H25	Age	Social influence	.001 (−.130 to .132)	.99
H26	Past use	Social influence	.062 (−.068 to .192)	.35
H27	Curiosity	Social influence	.313^b^ (.187 to .439)	<.001
H28	Price willing to pay	Social influence	.121 (−.010 to .253)	.07
H29	Age	Facilitating conditions	−.103 (−.233 to .026)	.12
H30	Past use	Facilitating conditions	.138^c^ (.010 to .266)	.04
H31	Curiosity	Facilitating conditions	.228^b^ (.100 to .357)	<.001
H32	Price willing to pay	Facilitating conditions	.207^b^ (.077 to .336)	.002
H33	Social influence	Perceived ease of use	.098 (−.043 to .238)	.17
H34	Social influence	Perceived usefulness	.253^b^ (.145 to .362)	<.001
H35	Social influence	Perceived enjoyment	.112 (−.002 to .226)	.05
H36	Social influence	Attitude toward purchase	.113 (−.016 to .243)	.09
H37	Social influence	Attitude toward using	.212^b^ (.087 to .337)	.001
H38	Social influence	Intention to purchase	.165^b^ (.061 to .268)	.002
H39	Social influence	Intention to use	.180^b^ (.073 to .287)	.001
H40	Facilitating conditions	Perceived ease of use	.389^b^ (.255 to .523)	<.001
H41	Facilitating conditions	Perceived usefulness	.022 (−.104 to .148)	.73
H42	Facilitating conditions	Perceived enjoyment	.291^b^ (.170 to .413)	<.001
H43	Facilitating conditions	Attitude toward using	.090 (−.046 to .225)	.19
H44	Facilitating conditions	Attitude toward purchase	.260^b^ (.123 to .397)	<.001
H45	Facilitating conditions	Intention to use	.139^c^ (.023 to .255)	.02
H46	Facilitating conditions	Intention to purchase	.111 (−.002 to .224)	.05

a H: hypothesis.

b *P*<.01*.*

c *P*<.05.

Curiosity influenced social influence (*P*<.001) and facilitating conditions (*P*<.001) but not perceived ease of use (*P*=.44), as originally hypothesized (Figure 2 and Table 2). Price willing to pay influenced perceived usefulness (*P*=.005) and facilitating conditions (*P*=.002). There was no relationship between price willing to pay and perceived ease of use, perceived enjoyment, or social influence (Figure 2 and Table 2). Age and past use did not predict any outcomes, with the exception of a relationship between past use and facilitating conditions (*P*=.04; Figure 2 and Table 2). Social influence was a predictor of perceived usefulness (*P*<.001), attitude toward using (*P*=.001), intention to purchase (*P*=.002), and intention to use (*P*=.001). There was no relationship between social influence and perceived ease of use, perceived enjoyment, or attitude toward purchase. Facilitating conditions influenced perceived ease of use (*P*<.001), perceived enjoyment (*P*<.001), attitude toward purchasing (*P*<.001), and intention to use (*P*=.02) but did not influence perceived usefulness, attitude toward using, or intention to purchase (Figure 2 and Table 2).

### Secondary Outcome

All scales demonstrated acceptable reliability, as determined by Cronbach α and composite reliability values. Cronbach α results ranged from .753 to .962, and composite reliabilities ranged from .756 to .962 (Table 3).

**Table 3.** Reliability of the measurement tools^a^.

Variable	Cronbach α	Composite reliability
Perceived usefulness	.952	0.952
Perceived ease of use	.915	0.916
Perceived enjoyment	.924	.918
Intention to use	.951	.952
Intention to purchase	.876	.877
Curiosity	.753	.756
Attitude toward using	.937	.937
Attitude toward purchasing	.962	.962
Social influence	.865	.861
Facilitating conditions	.818	.828

a The third item in the curiosity scale was deleted in the data analysis because it affects the reliability (if not deleted, Cronbach α would be .578), and the loading of this item is very low in confirmatory factor analysis (ie, the relationship between this item and the curiosity factor is weak).

## Discussion

### Principal Findings

The TAM-UTAUT predicted behavioral intentions associated with clinical VR use within this group of GME trainees. Predictors of intention to purchase include attitude toward purchasing, perceived enjoyment, perceived usefulness, and social influence, while predictors of intention to use include attitude toward using, perceived enjoyment, social influence, and facilitating conditions. Contrary to our hypothesis, perceived ease of use was not a predictor of intention to use. However, perceived ease of use predicted perceived enjoyment and attitude toward using; these latter 2 elements are effective mediators between perceived ease of use and intention to use.

The TAM-UTAUT also demonstrated that perceived enjoyment directly predicted future behavioral intentions to purchase and use VR technologies among GME trainees, but attitude toward using may not be a necessary mediator between perceived enjoyment and intention to use. Perceived enjoyment was a negative predictor of attitude toward purchase but a positive predictor of intention to purchase. It is possible that perceived usefulness and perceived ease of use constitute stronger justification to purchase a novel technology such as VR, whereas perceived enjoyment may not represent a compelling rationale to purchase a technology, even if behavioral intentions are predicted.

The VR-TAM that informed the tested model predicted that curiosity, age, past use, and price willing to pay all influenced perceived ease of use in the consumer market [21]. In contrast, this population of early career physicians indicated that curiosity, age, past use, and price willing to pay were not predictors of perceived ease of use. In addition, age and past use did not predict perceived usefulness. These results suggest that the previously hypothesized barriers to new technology adoption, such as older age or lack of prior exposure, do not affect GME trainees’ perceptions of ease of use or usefulness. The majority of trainees were 40 years or younger of age and had previous VR experience compared to the older mean age of faculty physicians. Given that GME trainees are more likely to be on the adoptive side of the “digital divide,” personal characteristics are less influential factors for predicting technology use [41].

This TAM-UTAUT demonstrated that price willing to pay was a predictor of perceived usefulness and facilitating conditions. These domains acted as mediators for predicting attitudes toward purchasing and using and intention to use, with implications for future adoption. VR is relatively affordable compared to other anxiolytics, especially as commercial equipment costs continue to decrease and health care VR applications expand [9,42-44]. As the difference between cost and price willing to pay continues to downtrend, there will be an increasing financial justification to apply VR in clinical settings from the perspective of GME trainees.

The UTAUT model elements, social influence and facilitating conditions, were hypothesized to predict behavioral intention to use [23]. The tested model indicated that social influence predicted perceived usefulness, attitude toward using, intention to purchase, and intention to use, while facilitating conditions predicted perceived ease of use, perceived enjoyment, attitude toward purchasing, and intention to use. These results further support these elements as key predictors for intention to use a novel technology [23]. However, in contrast to earlier studies where facilitating conditions was a stronger predictor of adoption than social influence, this TAM-UTAUT demonstrated social influence to be the stronger predictor [23,24]. In addition, this model indicates that social influence predicted intentions to both use and purchase, whereas facilitating conditions only predicted intention to use. One explanation for this incongruity may be that social influence and facilitating conditions are more context-dependent; different settings may engender different relationships between these extrinsic factors and technology adoption. Given that social influence and facilitating conditions are modifiable environmental factors, the positive directionality of their effects on behavioral intentions has strategic implications. Program directors and faculty responsible for educating GME trainees should foster learning environments that provide exposure to new technologies, relevant training and technical assistance, and social encouragement to drive VR adoption.

### Theoretical Implications

This TAM-UTAUT continues to expand the application of theoretical frameworks of technology adoption in health care and builds upon previous research by evaluating age, price willing to pay, and past use as continuous variables rather than categorical variables [24]. Additionally, with respect to the secondary aim, all TAM-UTAUT scales demonstrated good construct validity and reliability. This contributes to a growing body of evidence that TAMs and UTAUTs are appropriate modeling techniques to characterize technology adoption within clinical settings. This model also demonstrates the benefits of a hybrid model approach, as social influence and facilitating conditions from the UTAUT model were both predictors of intention to use. Future models can include other variables from the UTAUT model, such as performance expectancy and effort expectancy, as appropriate to the context and technology being studied.

### Limitations

There were several limitations to this investigation. First, trainees interested in VR may have been more likely to volunteer, leading to a selection bias. Second, participants were not involved in application setup or equipment troubleshooting, possibly leading to overestimated perceived ease of use. Third, factors such as perceived ease of use for patients and perceived usefulness for patients may affect attitudes to VR adoption. Future studies should explore patient perceptions as additional elements. Fourth, GME trainees were analyzed as a single group. A larger sample would have allowed for specialty subgroup analysis. Fifth, given the proximity to several notable technology company headquarters, participants may have stronger subjective norms toward VR compared to trainees in other regions. Similarly, the care settings in which this study was conducted already have established VR programs integrated into various aspects of patient care. While UTAUT scales like facilitating conditions attempt to characterize this context, this technology-driven culture may also have inflated other measures, such as perceived ease of use and perceived usefulness. To improve generalizability, additional studies should enroll trainees in other geographic settings or less technology-saturated regions.

### Conclusions

This TAM-UTAUT demonstrated validity and reliability for predicting the behavioral intentions of GME trainees to use VR as a therapeutic anxiolytic tool in clinical practice. Perceptions of usefulness, ease of use, and enjoyment predicted intention to use VR. Age, past use, price willing to pay, and curiosity were less strong predictors of intention to use. Social influence and facilitating conditions also strongly predicted behavioral intentions, representing opportunities to advance VR adoption.

## Supplementary material

10.2196/60767 Multimedia Appendix 1 Technology Acceptance Model or United Theory of Acceptance and Use of Technology survey.

10.2196/60767 Multimedia Appendix 2 Variables and items.

10.2196/60767 Multimedia Appendix 3 Participant specialties.

10.2196/60767 Multimedia Appendix 4 Summary of variables.

10.2196/60767 Multimedia Appendix 5 Standardized factor loadings in confirmatory factor analysis.

10.2196/60767 Multimedia Appendix 6 Confirmatory factor analysis of measures.

## Abbreviations

CFA: confirmatory factor analysis
CFI: comparative fit index
GME: graduate medical education
RA: research assistant
REDCap: Research Electronic Data Capture
RMSEA: root mean square error of approximation
SRMR: standardized root mean square residual
TAM: Technology Acceptance Model
TLI: Tucker-Lewis index
UTAUT: United Theory of Acceptance and Use of Technology
VR: virtual reality
