# Supplementary material for: Acceptance of Virtual Reality in Trainees Using a Technology Acceptance Model: Survey Study
Source: JMIR Med Educ. 2024 Dec 23;10:e60767. doi: 10.2196/60767 (PMC11693781; doi:10.2196/60767)
Supplement: Multimedia Appendix 1 [file mededu-v10-e60767-s001.docx]

Score the following statements on a scale from 1-5, 1= Strongly Disagree and 5= Strongly Agree

| *Perceived usefulness* |
| --- |
| I believe using VR hardware would help me be more productive in the hospital. |
| I believe using VR hardware would help me be more effective when providing patient care. |
| Using VR hardware would be useful in my work life. |
| Using VR hardware would improve my work life. |
| Using VR hardware would enhance my effectiveness in healthcare. |
| *Perceived ease of use* |
| I believe using VR hardware would be easy for me. |
| I believe it would be easy to get VR hardware to do what I want it to do. |
| I believe using VR hardware would be clear and understandable. |
| I would find VR hardware flexible to interact with. |
| It would be easy for me to become skillful at using VR hardware in the hospital. |
| *Perceived enjoyment* |
| I believe I would find using VR hardware enjoyable with patients. |
| I believe I would have fun using VR hardware with patients. |
| Using VR hardware would be exciting for patients. |
| Using VR hardware would be enjoyable for patients. |
| *Intention to use* |
| There is a high likelihood that I will use VR hardware within the foreseeable future with patients if I had access to it. |
| I intend to use VR hardware within the foreseeable future with patients if I had access to it. |
| I will use VR hardware within the foreseeable future with patients given access. |
| Using VR hardware in the foreseeable future is important to for me and my patients. |
| *Intention to purchase* |
| There is a high likelihood that I would support the hospital purchasing VR hardware within the foreseeable future. |
| I intend to support the hospital's procurement of VR hardware within the foreseeable future. |
| I will ask the hospital to purchase VR hardware within the foreseeable future. |
| The hospitals purchasing of VR hardware in the foreseeable future is important to me. |
| *Curiosity* |
| I like to shop around and look at displays. |
| I often read advertisements just out of curiosity. |
| Reading internet advertising to find out what's new is a waste of time. |
| I like to browse through catalogs or online stores even when I don't plan to buy anything. |
| *Social Influence* |
| People who influence my behavior think that I should use the VR system. |
| People who are important to me think that I should use the VR system. |
| Patients welcome me using the VR system. |
| Other colleagues welcome me using the VR system. |
| *Facilitating Conditions* |
| I have the resources necessary to use the VR system. |
| I will use VR if I receive appropriate training. |
| I will use VR if I receive the necessary technical assistance. |
| Given the resources, opportunities and knowledge it takes to use the VR system, it would be easy for me to use the VR system. |
| *Attitude toward using VR hardware (5-point scale)* |
| My impression of using VR hardware in the hospital setting is: |
| Bad - Good |
| Negative – Positive |
| Unsatisfactory - Satisfactory |
| Unfavorable - Favorable |
| Unpleasant - Pleasant |
| *Attitude toward purchasing VR hardware (5-point scale)* |
| My impression of the hospital purchasing VR hardware is: |
| Bad - Good |
| Negative – Positive |
| Unsatisfactory - Satisfactory |
| Unfavorable - Favorable |
| Unpleasant - Pleasant |
| *Price willing to pay* |
| Price you think the hospital should be willing to pay for a single VR device: |
| $ 0 - $1500 (continuous scale, $15 increments) |
