# Supplementary material for: Acceptance of Virtual Reality in Trainees Using a Technology Acceptance Model: Survey Study
Source: JMIR Med Educ. 2024 Dec 23;10:e60767. doi: 10.2196/60767 (PMC11693781; doi:10.2196/60767)
Supplement: Multimedia Appendix 2 [file mededu-v10-e60767-s002.docx]

| Variable | Items | Answers |
| --- | --- | --- |
| Perceived Usefulness | pu_1 – pu_5 | 1-5 |
| Perceived Ease of Use | peou_1 – peou_5 | 1-5 |
| Perceived enjoyment | pe_1 – pe_4 | 1-5 |
| Intention to use | itu_1 – itu_4 | 1-5 |
| Intention to purchase | itp_1 – itp_4 | 1-5 |
| Curiosity | c_1, c_2, c_4 | 1-5 |
| Attitude toward using | bad_good, pos_neg, sat_uns, fav_unf, unp_ple | 1-5 |
| Attitude toward purchasing | bad_good2, pos_neg2, sat_uns2, fav_unf2, unp_ple2 | 1-5 |
| Social influence | social_1 - social_4 | 1-5 |
| Facilitating condition | facilitating_1- facilitating_4 | 1-5 |
| Price willing to pay | cost($) | Continuous (0-1500) |
| Age | age | Continuous |
| Past Use | times_vr | Continuous (0-20) |
| Gender | sex | male, female, choose not to disclose |
