# Supplementary material for: Acceptance of Virtual Reality in Trainees Using a Technology Acceptance Model: Survey Study
Source: JMIR Med Educ. 2024 Dec 23;10:e60767. doi: 10.2196/60767 (PMC11693781; doi:10.2196/60767)
Supplement: Multimedia Appendix 3 [file mededu-v10-e60767-s003.docx]

| Specialty | Number of Respondents |
| --- | --- |
| Abdominal Imaging Radiology | 4 |
| Anesthesiology | 7 |
| Cardiothoracic Radiology, Diagnostic Radiology | 1 |
| Cardiovascular Disease, Internal Medicine | 3 |
| Child and Adolescent Psychiatry, Psychiatry | 2 |
| Child Neurology/Pediatric Neurology, Neurology | 4 |
| Clinical Biochemical Genetics, Medical Genetics and Genomics | 1 |
| Clinical Informatics, Diagnostic Radiology | 1 |
| Critical Care Medicine, Anesthesiology | 3 |
| Critical Care Medicine, Emergency Medicine | 1 |
| Critical Care Medicine, Internal Medicine | 5 |
| Diagnostic Radiology | 7 |
| Emergency Medicine | 3 |
| General Surgery | 12 |
| Gynecologic Oncology, Obstetrics and Gynecology | 1 |
| Hematology and Medical Oncology, Internal Medicine | 4 |
| Integrated Plastic Surgery | 1 |
| Internal Medicine | 27 |
| Internal Medicine-Critical Care Medicine, Emergency Medicine | 1 |
| Interventional Radiology, Diagnostic Radiology | 1 |
| Medical Genetics and Genomics | 1 |
| Musculoskeletal Imaging Radiology, Diagnostic Radiology | 2 |
| Neurocritical Care, Neurology | 1 |
| Neurological Surgery | 1 |
| Neurology | 8 |
| Neuroradiology, Diagnostic Radiology | 1 |
| Obstetrics and Gynecology | 4 |
| Ophthalmology | 2 |
| Orthopaedic Surgery | 2 |
| Other | 1 |
| Otolaryngology-Head and Neck Surgery | 6 |
| Pathology | 20 |
| Pediatric Cardiology, Pediatrics | 7 |
| Pediatric Critical Care Medicine, Pediatrics | 3 |
| Pediatric Nephrology, Pediatrics | 2 |
| Pediatric Radiology, Diagnostic Radiology | 1 |
| Pediatrics | 32 |
| Plastic Surgery | 2 |
| Psychiatry | 1 |
| Pulmonary Disease and Critical Care Medicine, Internal Medicine | 2 |
| Radiation Oncology, Diagnostic Radiology | 4 |
| Thoracic Surgery/Thoracic and Cardiac Surgery | 2 |
| Urology | 3 |
| Vascular Surgery, General Surgery | 1 |
