# Supplementary material for: Acceptance of Virtual Reality in Trainees Using a Technology Acceptance Model: Survey Study
Source: JMIR Med Educ. 2024 Dec 23;10:e60767. doi: 10.2196/60767 (PMC11693781; doi:10.2196/60767)
Supplement: Multimedia Appendix 4 [file mededu-v10-e60767-s004.docx]

|  | Minimum | Maximum | Mean | Std. Deviation |
| --- | --- | --- | --- | --- |
| pu_avg * | 1 | 5 | 3.498 | 0.883 |
| peou_avg | 1 | 5 | 3.911 | 0.803 |
| pe_avg | 1 | 5 | 4.349 | 0.721 |
| itu_avg | 1 | 5 | 3.460 | 1.064 |
| itp_avg | 1 | 5 | 3.446 | 0.880 |
| c_avg | 1 | 5 | 3.404 | 0.908 |
| att_use_avg | 2 | 5 | 3.979 | 0.751 |
| att_pur_avg | 2 | 5 | 3.810 | 0.801 |
| social_influence_avg | 1 | 5 | 2.910 | 0.729 |
| facilitating_cond_avg | 1 | 5 | 3.684 | 0.707 |
| Age | 23 | 45 | 31.313 | 3.508 |
| Past Use | 0 | 20 | 2.702 | 4.454 |
| Price willing to pay | 0 | 1500 | 781.364 | 375.459 |

*Note*. * Average Scores of the Scales
