# Supplementary material for: Acceptance of Virtual Reality in Trainees Using a Technology Acceptance Model: Survey Study
Source: JMIR Med Educ. 2024 Dec 23;10:e60767. doi: 10.2196/60767 (PMC11693781; doi:10.2196/60767)
Supplement: Multimedia Appendix 5 [file mededu-v10-e60767-s005.docx]

| Items | Loading |
| --- | --- |
| **Perceived Usefulness** |  |
| *I believe using VR hardware would help me be more productive in the hospital.* | .879 |
| *I believe using VR hardware would help me be more effective when providing patient care.* | .878 |
| *Using VR hardware would be useful in my work life.* | .915 |
| *Using VR hardware would improve my work life.* | .910 |
| *Using VR hardware would enhance my effectiveness in healthcare.* | .882 |
| **Perceived Ease of Use** |  |
| *I believe using VR hardware would be easy for me.* | .728 |
| *I believe it would be easy to get VR hardware to do what I want it to do.* | .780 |
| *I believe using VR hardware would be clear and understandable.* | .875 |
| *I would ﬁnd VR hardware ﬂexible to interact with.* | .882 |
| *It would be easy for me to become skillful at using using VR hardware in the hospital.* | .885 |
| **Perceived enjoyment** |  |
| *I believe I would ﬁnd using VR hardware enjoyable with patients.* | .703 |
| *I believe I would have fun using VR hardware with patients.* | .798 |
| *Using VR hardware would be exciting for patients.* | .927 |
| *Using VR hardware would be enjoyable for patients.* | .950 |
| **Intention to use** |  |
| *There is a high likelihood that I will use VR hardware within the foreseeable future with patients if I had access to it.* | .908 |
| *I intend to use VR hardware within the foreseeable future with patients if I had access to it.* | .928 |
| *I will use VR hardware within the foreseeable future with patients given access.* | .962 |
| *Using VR hardware in the foreseeable future is important to for me and my patients.* | .850 |
| **Intention to purchase** |  |
| *There is a high likelihood that I would support the hospital purchasing VR hardware within the foreseeable future.* | .580 |
| *I intend to support the hospital's procurement of VR hardware within the foreseeable future.* | .609 |
| *I will ask the hospital to purchase VR hardware within the foreseeable future.* | .892 |
| *The hospitals purchasing of VR hardware in the foreseeable future is important to me.* | .952 |
| **Curiosity** |  |
| *I like to shop around and look at displays.* | .683 |
| *I often read advertisements just out of curiosity.* | .739 |
| *I like to browse through catalogs or online stores even when I don't plan to buy anything.* | .715 |
| **Attitude toward using** |  |
| *Bad - Good* | .879 |
| *Negative - Positive* | .847 |
| *Unsatisfactory - Satisfactory* | .843 |
| *Unfavorable - Favorable* | .936 |
| *Unpleasant - Pleasant* | .806 |
| **Attitude toward purchasing** |  |
| *Bad - Good* | .896 |
| *Negative - Positive* | .915 |
| *Unsatisfactory - Satisfactory* | .929 |
| *Unfavorable - Favorable* | .939 |
| *Unpleasant - Pleasant* | .896 |
| **Social influence** |  |
| *People who influence my behavior think that I should use the VR system* | .570 |
| *People who are important to me think that I should use the VR system.* | .621 |
| *Patients welcome me using the VR system.* | .905 |
| *Other colleagues welcome me using the VR system* | .861 |
| **Facilitating condition** |  |
| *I have the resources necessary to use the VR system.* | .374 |
| *I will use VR if I receive appropriate training.* | .941 |
| *I will use VR if I receive the necessary technical assistance.* | .891 |
| *Given the resources, opportunities and knowledge it takes to use the VR system, it would be easy for me to use the VR system.* | .809 |
