# Supplementary material for: Acceptance of Virtual Reality in Trainees Using a Technology Acceptance Model: Survey Study
Source: JMIR Med Educ. 2024 Dec 23;10:e60767. doi: 10.2196/60767 (PMC11693781; doi:10.2196/60767)
Supplement: Multimedia Appendix 6 [file mededu-v10-e60767-s006.docx]

| Variable | CFI | TLI | RMSEA | SRMR |
| --- | --- | --- | --- | --- |
| Perceived usefulness | .985 | .970 | .123 | .014 |
| Perceived ease of use | .990 | .980 | .085 | .017 |
| Perceived enjoyment | 1 | 1 | 0 | .003 |
| Intention to use | .991 | .974 | .137 | .011 |
| Intention to purchase | .995 | .972 | .114 | .007 |
| Curiosity | 1 | 1 | 0 | 0 |
| Attitude toward using | .989 | .974 | .108 | .014 |
| Attitude toward purchasing | .984 | .968 | .137 | .013 |
| Social influence | 1 | 1 | 0 | 0.001 |
| Facilitating conditions | .994 | .983 | .081 | .021 |

*Note*: Since the curiosity scale has only three items, the CFA model is saturated and the model fitting is perfect and of little use.
